# Autism Spectrum Disorder Genes: Disease-Related Networks and Compensatory Strategies

**DOI:** 10.3389/fnmol.2022.922840

**Published:** 2022-06-03

**Authors:** Hye Kyung Lim, Jong Hyuk Yoon, Minseok Song

**Affiliations:** ^1^Department of Life Sciences, Yeungnam University, Gyeongsan, South Korea; ^2^Neurodegenerative Diseases Research Group, Korea Brain Research Institute, Daegu, South Korea

**Keywords:** autism spectrum disorder, genetic mice model, pathophysiology, pharmacological restoration, genetic restoration

## Abstract

The mammalian brain comprises structurally and functionally distinct regions. Each of these regions has characteristic molecular mechanisms that mediate higher-order tasks, such as memory, learning, emotion, impulse, and motor control. Many genes are involved in neuronal signaling and contribute to normal brain development. Dysfunction of essential components of neural signals leads to various types of brain disorders. Autism spectrum disorder is a neurodevelopmental disorder characterized by social deficits, communication challenges, and compulsive repetitive behaviors. Long-term genetic studies have uncovered key genes associated with autism spectrum disorder, such as SH3 and multiple ankyrin repeat domains 3, methyl-CpG binding protein 2, neurexin 1, and chromodomain helicase DNA binding protein 8. In addition, disease-associated networks have been identified using animal models, and the understanding of the impact of these genes on disease susceptibility and compensation is deepening. In this review, we examine rescue strategies using key models of autism spectrum disorder.

## Introduction

Autism spectrum disorder (ASD) is a clinically heterogeneous and inherited neurodevelopmental disorder. To diagnose a broad range of clinical phenotypes of the disease, the American Psychiatric Association published the Diagnostic and Statistical Manual of Mental Disorders (Bienvenu et al., 2013). Following the criteria indicated therein, ASD can be diagnosed on the basis of core manifestations of impaired social communication and interaction and restricted, repetitive behaviors, in addition to the different degrees of concurrent conditions, including intellectual deficit, language difficulty, epilepsy, sleep disturbance, and attention disability. According to the Centers for Disease Control and Prevention, the current overall prevalence of ASD is estimated to be 1 in 44 children, and there are 4.2 times more boys than girls with ASD (Maenner et al., 2021). Despite multi-faceted research being conducted to understand the molecular mechanism of ASD, its precise etiology remains unknown; however, both genetic and environmental factors are considered to play a role. Despite decades of research on novel pharmacological treatments for core ASD symptoms, the number of medications that exhibit therapeutic effects that meet certain criteria is still limited, and there is insufficient evidence for their clinical use. Several large-scale drug development efforts for ASD based on basic science and molecular research have yielded mostly negative or ambiguous clinical trial outcomes. As a result, the existing authorized ASD treatment methods only address secondary symptoms, including restlessness and impatience.

Non-invasive imaging studies have revealed anatomical and functional alterations in the brain, associated with the disorder (Kemper and Bauman, 1998; Amaral et al., 2008; Courchesne et al., 2011). Brain hypergrowth in patients with ASD is associated with an unusually excess number of neurons in the prefrontal cortex (PFC), according to a small-scale preliminary study analyzing post-mortem prefrontal tissue in children with ASD. Although post-mortem and structural magnetic resonance imaging studies have made it difficult to establish a clear, generalized pathology for overall ASD, researchers have been able to uncover pathological abnormalities in the frontal lobe, amygdala, and cerebellum in many cases of ASD. The brains of children and adults with ASD have been shown to exhibit a variety of morphological and functional abnormalities (Sparks et al., 2002; Amaral et al., 2008). However, these defects have not been consistently identified in distinct patients with ASD and are rarely correlated with symptom severity. Genetic predisposition to ASD has been well documented through research in families and twins. Exome sequencing, which is being performed on a continuously increasing number of patient samples, has identified novel rare mutations relevant to ASD (Voineagu et al., 2011; O’Roak et al., 2012; De Rubeis et al., 2014). Researchers identifying the genetic and pathological etiologies of patients with major ASD have faced enormous difficulty owing to the high heterogeneity in the expression and severity of the core and associated symptoms. Based on genetic investigations to date, it has been predicted that approximately 1000 genes are implicated in ASD (O’Roak et al., 2012; De Rubeis et al., 2014); however, no single variants have been found to be strongly linked to ASD.

**FIGURE 1:**
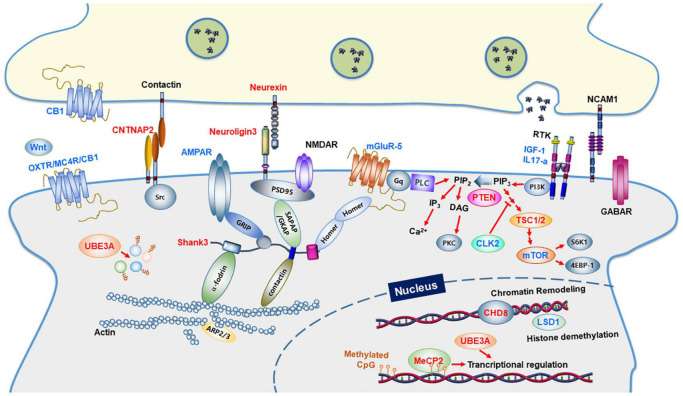
Synaptic proteins and signaling pathways linked to autism spectrum disorders. Synaptic proteins associated with ASD are involved in synapse formation and synaptic function. The ASD-linked proteins covered in this review article are highlighted in red. Proteins in blue indicate molecular targets that were used to ameliorate the ASD phenotype. CB1, Cannabinoid receptor type 1; OXTR, Oxytocin Receptor; MC4R, Melanocortin 4 Receptor; PLC, Phospholipase C; GABAR, GABAA receptor; AMPAR, AMPA receptor; NMDAR, NMDA receptor; mGluR, metabolic glutamate receptor; IGF-1, Insulin-like growth factor 1; IL17A, Interleukin 17A; NCAM1, Neural Cell Adhesion Molecule 1; GRIP1, Glutamate Receptor Interacting Protein 1; SHANK3, SH3 and multiple ankyrin repeat domains 3; SAPAP, Synapse-Associated Protein 90/Postsynaptic Density-95-Associated Protein; GKAP, guanylate-kinase-associated-protein; PSD-95, post-synaptic scaffolding protein 95 kDa; CNTNAP2, a contactin-associated protein-like 2 gene; PI3K, phosphoinositide-3 kinase; CLK2, CDC Like Kinase 2; PTEN, phosphatase and tensin homologs; TSC, tuberous sclerosis complex; mTOR, a mammalian target of rapamycin; CHD8, chromodomain helicase DNA binding protein 8; MeCP2, methyl CpG binding protein 2; LSD1, lysine-specific demethylase 1; UBE3A, Ubiquitin Protein Ligase E3A.

Because ASD has multigenic traits, understanding its etiology may be difficult. However, the related genes can be classified into several groups, allowing researchers to deduce the specific molecular pathways relevant to ASD (De Rubeis et al., 2014). ASD has been linked to genes involved in synaptic transmission and scaffolding, chromatin remodeling, protein synthesis and degradation, and actin cytoskeletal dynamics (Figure 1) (Bourgeron, 2015). Researchers must use experimental animal models to further understand how these genes are related to the etiology and pathophysiology of ASD. Mice are the most commonly used animal models in neuroscience. A number of characteristics explain why they are frequently used to describe human neuropsychiatric disorders. In reality, mice are social animals that live in hierarchical groups and engage in complex social activities, such as parenting and communal nesting of the young, juvenile play, and adult sexual and aggressive behaviors (Ellenbroek and Youn, 2016). They are usually selected because they can be used for neurobiological, behavioral, and pharmacological studies to examine autistic characteristics in humans and because of their rapid generation time, small size, and comprehensive genome sequencing (Wohr and Scattoni, 2013). Mice with a mutation in one of several ASD risk genes have been the most widely utilized animal models to date (Rubenstein and Merzenich, 2003; Lewis et al., 2007; Wohr and Scattoni, 2013; Ellenbroek and Youn, 2016; Chung et al., 2021). Behavioral tests used to evaluate social deficits, communication impairments, and repetitive activities have also been employed to validate the mouse models of ASD (Table 1).

**TABLE 1:** Patients’ phenotypes, as well as phenotypes and rescue strategies shown in ASD mouse models.

	Genes	Human phenotype	Mouse phenotype	Cellular phenotype	Rescue strategy	Rescued phenotype	References
Synaptic network	SHANK3	Speech-defective ASD, Phelan-McDermid syndrome, Schizophrenia	Core ASD symptoms, Anxiety	Hyperactive mGluR5 activity	MPEP (mGluR5 antagonist)	Grooming, Instrumental learning, Striatal synaptic plasticity	Wang et al., 2016
					LSD1 (H3K4me2 demethylase inhibitor)	Core symptoms	Rapanelli et al., 2022
					Rapamycin, CLK2 inhibitor, Akt activator, IGF-1	Social behavior	Bidinosti et al., 2016
					Adulthood correction with SHANK3 expression	Core symptoms	Mei et al., 2016
					IL-17a	Social behavior	Reed et al., 2020
	CNTNAP2 (NRXN4)	Speech-defective ASD, AR Pitt-Hopkins-like syndrom I	Core ASD symptoms	Projection neuron mismigration, Reduced GABAergic neurons and oxytocin neurons	Oxytocin	Social behavior	Penagarikano et al., 2015
					Ro27-3225 (melanocortin receptor 4 agonist)	Social behavior	Penagarikano et al., 2015
					CX546 (positive allosteric modulator of AMPA receptor)	Social interaction	Sacai et al., 2020
					IL-17a	Social behavior	Reed et al., 2020
					Risperidone	Repetitive behavior	Penagarikano et al., 2011
	NLGN3	GOF – X-linked ASD or Asperger syndrome	GOF – Impaired social interaction, KO – Fragile X syndrome	KO – Impaired corticostriatal gltamatergic synapses, E:I imbalance	Adulthood conditional correction with WT NLGN3 in the Purkinje cells	Synaptic plasticity	Baudouin et al., 2012
					Cannabinoid	Synaptic plasticity	Martella et al., 2018
Neuronal signaling	PTEN	ASD, Oncological syndromes	Impaired social interaction	Upregulation of the Akt-mTOR pathways, Downregulation of GSK3	Rapamycin (supression of mTORC1)	Social behavior	Zhou et al., 2009
					Reduced Rictor expression (suprression of mTORC2)	Social behavior	Zhou et al., 2009
	TSC1/TSC2	ASD, Tuberous sclerosis-1	Core ASD symptoms, Seizures	Increased number of astrocytes, Early increase of GABAergic interneurons	Rapamycin (supression of mTORC1)	Seizure, Social behavior	Zeng et al., 2008
	UBE3A	Maternal LOF – Angelman syndrome, Paternal duplication – ASD	Double or triple dose – Core ASD symptoms	Increased dose of UBE3A – Downregulation of glutamatergic synapse	Cbln1 genetic expression	Social interaction	Krishnan et al., 2017
Transcriptional and Chromatin Remodeling	CHD8	ASD (macrocephaly, distinct facial features, gastrointestinal motility)	Core ASD symptoms, Abnormal brain structure, Anxiety	Downregulation of Wnt signaling	Genetic restoration of β-catenin	Social behavior	Chahrour et al., 2008
	MECP2	ASD, Rett syndrome	Impaired social behavior, Anxiety, Learning and memory deficit	Impaired GABAergic neurons, Decrease of BDNF expression	GABA_*A*_ receptor agonist	Social behavior, Anxiety, but not Memory	Chang et al., 2006
					IGF-1	Motor function, Breathing pattern	Tanaka et al., 2012
					BDNF	Motor function, Electrophysiological deficits	Hogart et al., 2007
							

The hallmark characteristic of ASD is abnormal social interaction, and several behavioral tests can be used to assess social approach, reciprocal social interactions, nesting, sexual interactions, parenting behaviors, and hostile encounters (Wohr and Scattoni, 2013; Pensado-Lopez et al., 2020). An automated three-chambered social approach task with a stranger and non-social object on the opposite end can be utilized to reveal the deficiency of sociability based on the time spent during the task (Yang et al., 2011). The social approach task can also be used to measure social recognition by replacing stimuli with familiar and novel mice. An open field apparatus can be used for social and sexual interactions by placing two unfamiliar mice together to evaluate more sophisticated social behaviors. The mice interact primarily by sniffing, following, crawling, and pushing each other. Another ASD criterion is a deficit in communication, which is the most challenging assay because mice do not use language. Instead, mice produce ultrasonic vocalizations under various conditions. Such ultrasonic vocalizations have been found in newborns at the time of birth, pups removed from their mother, juveniles during social play, and adults during mating, aggression, or exploration (Scattoni et al., 2009). Restricted, repetitive, and stereotyped patterns of behavior are fundamental characteristics of ASD. Vertical jumping, backflipping, circling, digging, marble burying, rearing, and excessive self-grooming are common behaviors in mice (Lewis et al., 2007). The open field test measures the presence and duration of locomotor activity. Reversal learning tasks assess the capacity to adapt to a new routine in the T-maze or Morris water maze (Zaccaria et al., 2010). The tendency of mice to investigate novel objects and poke their nose into holes in the wall or floor can be used to reflect restricted interests (Moy et al., 2008).

Following the establishment of genetically engineered mouse models, behavioral research, biochemical studies, and neurophysiological investigations have validated the link between candidate genes and ASD phenotypes. Various studies using animal models of ASD are currently providing fundamental knowledge regarding the biology and treatment strategies of the disease. Based on the findings of analyses of signal circuits for ASD models, it is conceivable to provide molecular targets relevant for therapeutic intervention, yielding optimism that ASD caused by defects in several genes could be treated even after brain development has been completed to some extent. In this review, we explain the mouse models that can help in understanding ASD, the pathological characteristics noted from each model, and pharmacological restoration and potential rescue tactics derived for future clinical trials.

## Mouse Models

ASD-related mouse models are implicated in three generally defined genes: those engaged in the synaptic network, neuronal signaling, and transcriptional and chromatin remodeling, based on the discovered risk factors and biological etiology (De Rubeis et al., 2014; Yoo, 2015; Rylaarsdam and Guemez-Gamboa, 2019; Chung et al., 2021). Various mouse models with targeted mutations in these candidate genes have been developed and used to explore the pathophysiological mechanisms of ASD biology to better understand the genetics of ASD. Each mouse model provided novel insights into the pathophysiology of ASD, and pharmacological and genetic restoration experiments using these mouse models revealed important clues for future clinical trials.

### Synaptic Network

Mutations in synapse-related genes, such as SH3 and multiple ankyrin repeat domains (SHANK), neurexin (NRXN), contactin-associated protein-like 2 (CNTNAP2), and neuroligin (NLGN), are the most commonly described genetic etiologies in ASD. Abnormalities in these synaptic genes are linked to ASD and other complex neuropsychiatric illnesses, such as locomotor difficulties, intellectual impairments, seizures, and speech issues. Mouse models that target these synaptic genes have primarily been used to precisely understand the molecular mechanisms of ASD and have made significant contributions to the discovery of relevant neuronal pathways and plausible therapeutic approaches.

#### Neuroligin 3

Neuroligins (NLGNs) are post-synaptic adhesion proteins that serve as ligands for NRXNs and pre-synaptically localized adhesion molecules that support Ca^2+^-dependent trans-synaptic binding to initiate synaptogenesis at central synapses (Reissner et al., 2008). Among the five isoforms in humans, NLGN3 is highly associated with non-syndromic ASD. A gain-of-function mutation in NLGN3 (R451C) predisposes patients to X-linked ASD or Asperger syndrome (Jamain et al., 2003). Synaptic transmission and heterosynaptic competition have been reported to be disturbed in NLGN3 knockout mice owing to disruption in metabotropic glutamate receptor-dependent synaptic plasticity. Autism is a developmental disorder that is usually diagnosed after 3 years of age. Researchers have attempted to determine whether the behavioral traits of ASD can be reversed in adults with abnormally developed brain structures over long periods of time. A resolution to this topic was provided by rearing genetically modified mice with a tetracycline transactivator to temporally control the re-expression of wild-type NLGN3 in the Purkinje cells of NLGN3 knockout mice. The synaptic abnormalities observed in NLGN3 knockout mice could be reversed when the NLGN3 gene was restored after brain development was complete, indicating that the condition could be rescued even after a period of time had elapsed since its onset (Baudouin et al., 2012). The development of long-term synaptic depression at corticostriatal glutamatergic synapses was observed in R451C-NLGN3 mutant mice, corroborating the evidence for an imbalance between excitatory and inhibitory (E:I) neurotransmissions in models of ASD. Stimulation of the cannabinoid CB1 receptor partially relieved long-term synaptic depression in this model, suggesting that altered cannabinoid signaling may underpin the synaptic plasticity impairments observed in ASD (Martella et al., 2018). A recent optogenetic study in R451C knock-in mice demonstrated that social deficiencies in autism are caused by gamma oscillation disruption in the mPFC and that modulating mPFC PV interneurons even in adulthood may help correct the deficits (Cao et al., 2018).

#### Contactin-Associated Protein-Like 2

Contactin-associated protein-like 2 (CNTNAP2), also known as NRXN4, is an NRXN superfamily transmembrane protein found in the CNS. During nervous system development, it influences the subcellular localization of potassium channels within developing axons and mediates synapse formation between neurons and glia (Poliak et al., 1999). In the developing human brain, CNTNAP2 is abundant in circuits that are critical for language development. Genetic abnormalities in CNTNAP2 underlie speech-defective ASD and autosomal recessive Pitt–Hopkins-like syndrome-1, characterized by severe intellectual disability, regression of speech development, and behavioral abnormalities (Alarcon et al., 2008; Arking et al., 2008; Bakkaloglu et al., 2008; Smogavec et al., 2016). CNTNAP2 knockout mice display all the core behavioral characteristics of ASD, with cortical projection neuron migratory impairments, a decrease in GABAergic intermediate neurons, and concurrent altered brain synchrony (Penagarikano et al., 2011). In CNTNAP2 mutant mice, the number of oxytocin-positive neurons in the paraventricular nucleus is reduced, similar to brain oxytocin levels. Consistent with these findings, postnatal treatment with the neuropeptide oxytocin improves aberrant social behaviors in CNTNAP2 mutant mice (Penagarikano et al., 2015). Social deficiency has been successfully corrected in CNTNAP2 knockout mice upon treatment with a selective melanocortin receptor 4 agonist known to promote endogenous oxytocin release, whereas the oxytocin antagonist had the opposite effect. Based on these findings, it is plausible to deduce that in the CNTNAP2 knockout model, oxytocin signaling failure causes ASD-like behavior. CNTNAP2 has been found to be important in excitatory synaptic transmission and maintenance of E:I balance in pyramidal neurons of the developing mouse PFC. The impaired social interaction shown in CNTNAP2 mutant mice was significantly restored when AMPA receptor activity was enhanced through pharmacological treatment, suggesting that disrupted E:I balance in layer 2/3 pyramidal neurons of the PFC led to impaired social interaction in CNTNAP2 knockout mice (Sacai et al., 2020).

#### SH3 and Multiple Ankyrin Repeat Domains 3

SH3 and multiple ankyrin repeat domains (SHANK) is a large protein with several protein interaction domains, including ankyrin repeats, an SH3 domain, a PDZ domain, a proline-rich region, and an SAM domain (Sheng and Hoogenraad, 2007). The multidomain protein isoforms are generated by three SHANK genes that are differentially expressed in developmental stages, cell types, and brain regions (Boeckers et al., 1999; Bonaglia et al., 2001). SHANK family proteins play a critical role in the organization of post-synaptic molecular complexes in excitatory synapses through interactions with cytoskeletal regulators, endocytic machinery, diverse receptors, other adapters, and synaptic scaffold proteins (Naisbitt et al., 1999; Tu et al., 1999; Yao et al., 1999; Zitzer et al., 1999a,b; Kreienkamp et al., 2000; Tobaben et al., 2000; Lim et al., 2001; Bockers et al., 2004). Studies have shown that SHANK proteins play a vital role in dendritic spine formation and neurotransmission (Hering and Sheng, 2001; Sala et al., 2001; Ehlers, 2002; Rubenstein and Merzenich, 2003; Shcheglovitov et al., 2013; Gogolla et al., 2014; Filice et al., 2016; Lu et al., 2016; Yi et al., 2016).

SH3 and multiple ankyrin repeat domains 3 (SHANK3) deficiency in humans is associated with language and social communication problems of ASD, Phelan–McDermid syndrome, and schizophrenia (Durand et al., 2007; Gauthier et al., 2010). To further understand the molecular mechanisms underlying the role of SHANK3 in ASD, several research groups have developed SHANK3 mouse models that closely resemble human ASD characteristics, making a significant contribution to the understanding of the pathophysiology of ASD. SHANK3-deficient mice, both homozygous and heterozygous, displayed repetitive grooming, anxiety, and social interaction deficits (Bangash et al., 2011; Peca et al., 2011; Wang et al., 2011). SHANK3 gene deletion also affects the spine number and size, as well as AMPA transmission, indicating that SHANK proteins play a crucial role in excitatory neurotransmission. Indeed, follow-up studies have revealed that in the absence of SHANK3, excitatory input from the cortex to the striatum develops abnormally, resulting in an altered E:I ratio in the cortex and striatum (Peixoto et al., 2016).

A mouse model was employed to study the signaling circuit related to SHANK3 and played a key role in presenting the molecular network considered to be involved in ASD, in addition to presenting the anatomical and physiological characteristics that can arise in ASD. The findings have led to the development of a novel therapeutic strategy that was immediately verified using an animal model. SHANK3 deficiency causes the mGluR5-dependent corticostriatal-thalamic circuit to become hypoactive during social behaviors (Wang et al., 2016). In the mouse model, modulation of mGluR5 activity restored excessive grooming, instrumental learning, and striatal synaptic plasticity, suggesting that mGluR5 can be used to treat ASD. In the PFC of patients with ASD and SHANK3 mutant mice, histone lysine 4 demethylation (H3K4me2) was drastically reduced. Treatment of autism models with highly selective inhibitors of the H3K4me2 demethylase LSD1 results in a robust rescue of the core symptoms of ASD, revealing an important role of H3K4me2 abnormality in ASD pathophysiology and the therapeutic potential of targeting H3K4me2 demethylase LSD1 (Rapanelli et al., 2022). SHANK3-deficient neurons showed decreased ubiquitin-dependent degradation of CLK2 and impaired activation of Akt and mammalian target of rapamycin complex 1 (mTORC1) pathway proteins (Bidinosti et al., 2016). In Phelan–McDermid syndrome-derived neurons, pharmacological activation of Akt or inhibition of CLK2, and insulin-like growth factor-1 treatment restored excitatory synaptic activity in an Akt-dependent manner. In a SHANK3-deficient mouse model, CLK2 inhibition also restored normal sociability, providing novel mechanistic and potentially therapeutic insights into SHANK3-deficient signaling.

As previously stated, expressing wild-type NLGN3 after maturity can ameliorate the ASD phenotype in NLGN3-null mice. We tested whether the reversible features of ASD pathophysiology apply to an ASD model with other genetic abnormalities, using SHANK3 knockout mice. The answer was revealed by rearing transgenic mice with SHANK3 exons flipped upside down and then utilizing genetic manipulation to restore the normal orientation of the reverse exons once the mice had reached adulthood (Mei et al., 2016). Mice with inverted exons had biochemical, anatomical, and electrophysiological defects, and behavioral characteristics of ASD, which were confirmed to be substantially reversed when the exon was corrected. These findings suggest that aberrant development of the autistic brain caused by a congenital SHANK3 gene defect has considerable plasticity and can be substantially restored with later treatment.

Immune system dysregulation is seen in certain patients with ASD, and it is hypothesized that such a dysregulation could contribute to the development of ASD (Hsiao, 2013; Gottfried et al., 2015). Surprisingly, during systemic inflammation, some children with ASD appear to have improved autistic behavioral symptoms (Curran et al., 2007). When a mouse model deficient in CNTNAP2, FMR1, and SHANK3 was compared with an environmental model of a neurodevelopmental disorder in which mice were exposed to maternal immune activation during embryonic development, it was demonstrated that interleukin (IL)-17a production during inflammation directly affects the nervous system to improve the expression of social behavioral deficits (Reed et al., 2020). This research points to a unique neuroimmune mechanism underlying neurodevelopmental disorders, and IL-17a as a potential therapeutic target for autistic behavioral improvement.

### Neuronal Signaling

Downstream and upstream signaling pathways of synaptic receptors and channels influence synapse formation, development, and elimination. The signaling molecules control the size and characteristics of neurotransmission and therefore regulate gene expression programs in neurons. This gene group includes kinases, phosphatases, and adaptors, and other components of neuronal signaling, with phosphatase and tensin homolog (PTEN) and mammalian target of rapamycin (mTOR) signal complexes serving as examples.

#### Phosphatase and Tensin Homolog

Phosphatase and tensin homolog (PTEN) is a tumor suppressor gene that encodes dual-specificity phosphatase and suppresses the PI3K-mTOR signaling pathway (Luo et al., 2003). It regulates synaptogenesis, connections, and synaptic plasticity in the brain and is a critical modulator of synaptic function. PTEN deficiency is linked to oncological syndromes in humans, and social interaction and communication disorders, repetitive behaviors, and epilepsy in some circumstances (Rademacher and Eickholt, 2019). PTEN deficiency in the mouse CNS resulted in forebrain macrocephaly and neuronal hypertrophy, aberrant dendritic and axonal development, and synapse density (Kwon et al., 2006; Zhou and Parada, 2009; Takeuchi et al., 2013). These morphological changes are linked to upregulation of the Akt-mTOR pathways and downregulation of GSK3 caused by PTEN dysfunction, resulting in aberrant social interactions and excessive responses to sensory stimuli (Kwon et al., 2006).

Rapamycin effectively inhibits the overactivation of mTORC1 in the mTOR pathway, thereby improving neuronal hypertrophy and behavioral abnormalities in PTEN knockout mice (Zhou et al., 2009). In PTEN-null mice, mTORC2, which is less sensitive to rapamycin, is also overactivated. The behavioral and neurophysiological abnormalities of PTEN-null mice were improved when Rictor expression was suppressed using an antisense oligonucleotide to selectively disrupt the function of mTORC2; this finding suggests that mTORC1 and mTORC2 hyperactivities are the primary driver of the neuropathophysiology associated with PTEN deficiency (Chen et al., 2019).

#### Tuberous Sclerosis 1

The tumor suppressor gene Tuberous Sclerosis 1 (TSC1) forms a molecular complex with tuberin (TSC2) to inactivate the mTOR pathway (van Slegtenhorst et al., 1997). TSC1/2 is intimately associated with ASD, and ASD-like symptoms can be seen in approximately 30–60% of patients with TSC (Numis et al., 2011; Bahl et al., 2013). To determine the role of TSC1/2 in ASD, researchers developed a mouse model and found that heterozygous TSC1/2 mice showed an increase in the number of astrocytes, implying that the TSC1/2 complex is an essential regulator of astrocyte growth (Uhlmann et al., 2002). Conditional knockout of TSC1 resulted in brain enlargement, progressive epilepsy, and premature death (Zeng et al., 2008). TSC1-haploinsufficient and -null mice displayed impaired social behaviors, as well as an early increase in terminal axonal branching and synaptic density of PV-positive GABAergic interneurons (Amegandjin et al., 2021). These findings indicate that alterations in the mTOR signaling pathway caused by TSC1/2 mutations may be linked to the development of ASD, with the aim of determining whether rapamycin can improve ASD symptoms. In heterozygous or homozygous TSC1 mutant mice, rapamycin administration suppressed mTOR, resulting in reduced S6 phosphorylation, and alleviated aberrant social behaviors and seizures (Zeng et al., 2008; Tsai et al., 2012; Abs et al., 2013).

#### Ubiquitin-Protein Ligase E3A

Ubiquitin-Protein Ligase E3A (UBE3A) acts as an E3 ligase in the ubiquitin-proteasome pathway and is engaged in synaptic plasticity as well as transcriptional coactivation (Scheffner et al., 1993; Nawaz et al., 1999; Margolis et al., 2010; Sun et al., 2015). Angelman syndrome is caused by a maternal genetic loss of function of chromosome 15q containing the UBE3A gene, which is characterized by severe neurological and motor impairments (Albrecht et al., 1997; Cook et al., 1997). Transgenic mice with increased UBE3A levels, which show deficiencies in social interactions and stereotyped repetitive behaviors, were reared to investigate the function of this gene in brain developmental disorders (Smith et al., 2011). In UBE3A transgenic mice, there was a decrease in excitatory synaptic transmission owing to a reduction in the probability of presynaptic glutamate release and suppression of the post-synaptic action potential (Smith et al., 2011). Social behavioral defects were induced when UBE3A was overexpressed in the midbrain ventral tegmental area owing to downregulation of the glutamatergic synaptic organizer Cbln1, which is required for sociability; however, this was resolved by genetically restoring UBE3A in glutamatergic neurons in the region (Krishnan et al., 2017). The E:I balance in the mPFC of the Angelman syndrome model was considerably disrupted, and the interneuron excitability decreased. Increased Arc expression and decreased AMPA subtypes of glutamate receptors at excitatory synapses result from UBE3A deletion (Greer et al., 2010). Even when the brain is fully mature, physiological alterations in the mPFC caused by a defect in the UBE3A gene have been shown to be fully reversible upon gene reactivation; this implies that there is no substantial developmental window for reversing the physiological deficiencies detected in UBE3A conditional knockout mice (Rotaru et al., 2018).

### Transcriptional and Chromatin Remodeling

Transcriptional and chromatin remodeling are other categories of genes linked to ASD. ATP-dependent chromatin remodelers and modifiers are crucial for chromatin control (Ronan et al., 2013). Chromatin remodelers adjust the status of nucleosomes to coordinate gene transcription, whereas chromatin modifiers add or remove acetylation, methylation, ubiquitylation, and phosphorylation of histone proteins. These mechanisms, which allow neuronal growth, differentiation, and connectivity, are regulated by chromatin remodeling (Ronan et al., 2013).

#### Chromodomain Helicase DNA Binding Protein 8

Chromodomain Helicase DNA Binding Protein 8 (CHD8) is an ATP-dependent chromatin remodeling protein that directly recruits and binds β-catenin from the promoter region of the β-catenin target gene, thereby repressing β-catenin-mediated transcription (Thompson et al., 2008). Disruptive CHD8 mutations are strongly linked to ASD and are characterized by macrocephaly, distinct facial features, and gastrointestinal motility (Bernier et al., 2014). ASD-like symptoms, including social behavioral problems, repetitive activities, and increased anxiety, are observed in heterozygous individuals with CHD8 mutations (Katayama et al., 2016). CHD8 knockdown resulted in the downregulation of several Wnt signaling pathway transducers and effectors, including FZD1, FZD2, DVL2, DVL3, and CTNNB1, leading to abnormal neural progenitor cell proliferation and differentiation during cortical development. Furthermore, CHD8 knockdown resulted in abnormalities in brain structure, increased anxiety levels, and aberrant social interactions in adult mice (Durak et al., 2016). Finally, whether augmenting Wnt signaling could alleviate the ASD phenotype caused by CHD8 knockdown was examined. *In utero* electroporation was used to co-express a β-catenin construct with CHD8 shRNA at E13. Genetic restoration of β-catenin rescued the anatomical, developmental, and behavioral defects caused by CHD8 knockdown, suggesting that the ASD-related phenotype induced by CHD8 dysfunction can be improved by enhancing the Wnt signaling pathway (Thompson et al., 2008).

#### Methyl-CpG Binding Protein 2

Methyl-CpG Binding Protein 2 (MECP2) is a chromatin-associated protein that binds to methylated CpG and has a role in transcriptional activation or repression of possibly hundreds of genes (Nan et al., 1997; Chahrour et al., 2008). It is an X-linked gene necessary for neural maturation in both mice and humans during embryonic development (Tate et al., 1996). MECP2 inhibits the expression of BDNF by specifically regulating BDNF promoter III (Chen et al., 2003). After depolarization, neurons release more BDNF owing to the dissociation of the MECP2-histone deacetylase-mSin3A inhibitory complex from the promoter due to reduced CpG methylation, indicating that MECP2 plays a key role in BDNF expression (Martinowich et al., 2003). It is clear from these findings that DNA methylation and related chromatin remodeling in response to neural activity play a significant role in gene transcription regulation (Chen et al., 2003; Martinowich et al., 2003). Rett syndrome and ASD, which are progressive neurodevelopmental diseases marked by an increase in repetitive stereotyped behaviors and loss of social, cognitive, and language abilities, have a high prevalence of MECP2 gene mutations (Zappella et al., 2001; Heindel et al., 2006; Swanberg et al., 2009).

Methyl-CpG Binding Protein 2 (MECP2)-null mice showed impaired nest-building activity, learning and memory deficits, and defective social interaction (Moretti et al., 2005). Deletion of MECP2 in forebrain GABAergic neurons recapitulated Rett syndrome and ASD symptoms, such as repetitive behaviors, impaired motor coordination, and altered sensorimotor arousal (Chao et al., 2010). In MECP2 mutant mice, tactile over-reactivity was reduced by restricted treatment with a GABA_*A*_ receptor agonist in peripheral mechanosensory neurons, and chronic treatment improved anxiety-like behaviors and social impairments, but not memory deficits (Orefice et al., 2019). Insulin-like growth factor 1 is a pleiotropic growth factor that acts similarly to BDNF in the nervous system, stimulating the PI3K/pAkt/PSD-95 and MAPK pathways and increasing excitatory post-synaptic currents (Zheng and Quirion, 2004; Ramsey et al., 2005). Insulin-like growth factor-1 treatment normalized spine density and synaptic amplitude, increased PSD-96, and stabilized cortical plasticity in MECP2 mutant mice, which led to partial improvements in motor function and breathing patterns (Tropea et al., 2009).

Furthermore, MECP2 mutant mice had lower BDNF expression in the brain, and conditional deletion of BDNF in MECP2 mutants accelerated the development of RTT-like syndrome (Chang et al., 2006). Motor impairments were restored, and electrophysiological deficits were alleviated when BDNF expression increased in MECP2 mutant mice (Chang et al., 2006). Interestingly, MECP2-deficient mice had a higher rate of neuronal BDNF secretion but a lower overall BDNF content, implying that MECP2 deficiency disrupts the balance between BDNF protein expression and secretion, impairing synaptic BDNF signaling (Wang et al., 2006).

According to several studies, MECP2 loss suppresses the expression of UBE3A and another autism-associated gene (GABRB3) in the postnatal mammalian brain (Samaco et al., 2005; Hogart et al., 2007; Tanaka et al., 2012). MECP2 loss causes epigenetic abnormalities, which result in increased antisense RNA levels and decreased UBE3A production, implying a mechanistic link between MECP2 and UBE3A-induced autism (Makedonski et al., 2005).

MECP2 transgenic mice overexpressing the human MECP2 gene displayed MECP2 replication syndrome symptoms, and by 10 weeks of age, they had increased contextual learning and motor function, and improved synaptic plasticity in the hippocampus (Collins et al., 2004). Consistent with the results of the SHANK3 knockout model, when MECP2 expression was genetically restored in adult MECP2-null mice, all neurological abnormalities were reversed, and the phenotype significantly improved; this finding implies that aberrant brain development caused by MECP2 loss can be addressed even after a certain amount of time (Guy et al., 2007).

## Discussion

Several investigations have been conducted on ASD, using genome sequencing to identify gene mutations in patients with ASD, non-invasive brain imaging studies, anatomical and neurophysiological studies, and most importantly, development and examination of animal models with candidate genes. Given the genetic manipulation processes used to create transgenic animals, generating the syndrome ASD model is rather straightforward and efficient. Since each gene associated with syndromic ASD is located in one of the principal signaling pathways in neurons, dysregulation of genes upstream or downstream of the pathway might cause similar abnormalities. As a result, the syndromic ASD model meets the requirements for representativeness and convenience in ASD research. However, it must be emphasized that research using the syndrome ASD model have limitations in presenting knowledge for non-syndromic ASD, which accounts for a high percentage of cases. So far, the etiology and pathophysiology of ASD remain poorly understood. The fact that the genetic causes of ASD are diverse is the primary reason for these difficulties. As ASD is caused by a variety of genetic abnormalities, even if effective medications are developed for one subtype of ASD, they may not be successful against another subtype of ASD. Furthermore, because the genetic causes in patients are diverse, it is possible to conclude that the effectiveness of a particular drug is low; this is because it is beneficial only in a portion of patients during the development phase. Consequently, this disease needs to be characterized more precisely to create medications for treatment and gaining better knowledge of the disease itself.

To date, E:I imbalance in cortical and subcortical neuronal circuits is one of the most prominent theories underlying ASD (Rubenstein and Merzenich, 2003). This theory is notable because it could explain the frequent observation of reduced GABAergic signaling in the developmental ASD brain (Cellot and Cherubini, 2014). Any defect in GABAergic inhibition increases noise in the cortex, basal ganglia, and hippocampus. Abnormal glutamatergic neurotransmission has also been reported in adult ASD brain regions, such as the primary sensorimotor cortex (He et al., 2021). Defects in the several genes mentioned above may cause alterations in the glutamatergic and GABAergic systems, resulting in an overall increase in the E:I ratio in ASD (Canitano and Palumbi, 2021). Glutamatergic hyperactivity is associated with NLGN, NRXN, and SHANK. Genetic defects in NLGN and NRXN reduce excitatory activity and the NMDA/AMPA ratio (Etherton et al., 2009). SHANK knockout results in impaired mGluR5-dependent modulation of neural network activity and reduced NMDA receptors, specifically at synapses (Verpelli et al., 2011). GABAnergic inhibitory dysfunction has been observed in MECP2 and CNTNAP2 deficiencies. IL-17a is expected to affect the firing pattern of certain neurons, which has been shown to improve behavioral abnormalities in animal models of ASD; however, further investigation is required to fully understand its therapeutic benefits. Based on these findings, it may be determined that a group of ASD-related genes causes the disease by disturbing the E:I balance. However, further research is necessary to determine if the deletion of these genes leads to a disturbance in the overall E:I balance owing to the impairment of the same type of neurons in the same brain region.

Furthermore, certain genes implicated in the development of ASD may cause the disease through a mechanism other than E:I imbalance. For example, the molecular mechanisms through which PTEN or mTOR complex components cause ASD are not fully understood. They may have a pathogenic mechanism distinct from E:I imbalance because they are engaged in the regulation of overall translation. Additionally, oxytocin is considered a promising treatment for ASD, although the precise mechanism of action is unclear, apart from the fact that it affects the limbic system to regulate sociability and anxiety. Therefore, distinct groups of ASD-associated genes may be involved in diverse neurophysiological features that are yet to be fully delineated, and it is difficult to predict the diversity of these genes.

Although the contribution of each gene to the development of ASD is small (less than 1%), it is determined that each gene is part of a distinct biochemical pathway and is engaged in the production of a common output (Voineagu et al., 2011). This finding suggests that therapeutics that involve biological pathways in which they converge, rather than individual genes, could be effective. Therapeutic medications discovered using this approach could be widely employed in the treatment of ASD caused by defects in a specific pathway, thereby benefiting a larger patient population.

In summary, ASD should be categorized according to the molecular pathways in which each disease gene converges, and common features and potential therapeutic targets for these groups should be determined through multi-faceted studies, including animal model studies. The number of ASD-associated genes in larger patient samples will continue to increase in the future. If researchers can demonstrate the molecular connections between ASD genes and identify their function in specific brain areas, ASD can be classified more effectively. This work can serve as the foundation for developing biomarkers for each subgroup and identifying therapeutic strategies.

## Author Contributions

HL, JY, and MS developed the writing plan and drafted the manuscript. HL and MS developed the figure. All authors approved the final manuscript.

## Conflict of Interest

The authors declare that the research was conducted in the absence of any commercial or financial relationships that could be construed as a potential conflict of interest.

## Publisher’s Note

All claims expressed in this article are solely those of the authors and do not necessarily represent those of their affiliated organizations, or those of the publisher, the editors and the reviewers. Any product that may be evaluated in this article, or claim that may be made by its manufacturer, is not guaranteed or endorsed by the publisher.

## Abbreviations

ASD: autism-spectrum disorder
DSM: the Diagnostic and Statistical Manual of Mental Disorders
CDC: Centers for Disease Control and Prevention
USVs: ultrasonic vocalizations
SHANK: SH3 and multiple ankyrin repeat domains
NLGN: neuroligins
LTD: long-term synaptic depression
PVN: paraventricular nucleus
PFC: prefrontal cortex
mTORC1: mTOR complex 1
CB1: Cannabinoid receptor type 1
OXTR: Oxytocin Receptor
MC4R: Melanocortin 4 Receptor
PLC: Phospholipase C
GABAR: GABAA receptor
AMPAR: AMPA receptor
NMDAR: NMDA receptor
mGluR: metabolic glutamate receptor
IGF-1: Insulin-like growth factor 1
IL17A: Interleukin 17A
NCAM1: Neural Cell Adhesion Molecule 1
GRIP1: Glutamate Receptor Interacting Protein 1
SAPAP: Synapse-Associated Protein 90/Postsynaptic Density-95-Associated Protein
GKAP: guanylate-kinase-associated-protein
PSD-95: post-synaptic scaffolding protein 95 kDa
CNTNAP2: a contactin-associated protein-like 2 gene
PI3K: phosphoinositide-3 kinase
CLK2: CDC Like Kinase 2
PTEN: phosphatase and tensin homologs
TSC: tuberous sclerosis complex
mTOR: a mammalian target of rapamycin
CHD8: chromodomain helicase DNA binding protein 8
MeCP2: methyl CpG binding protein 2
LSD1: lysine-specific demethylase 1
UBE3A: Ubiquitin Protein Ligase E3A.

## References

[B1] Abs E. Goorden S. M. Schreiber J. Overwater I. E. Hoogeveen-Westerveld M. Bruinsma C. F. (2013). TORC1-dependent epilepsy caused by acute biallelic Tsc1 deletion in adult mice. *Ann. Neurol.* 74 569–579. 10.1002/ana.23943 23720219

[B2] Alarcon M. Abrahams B. S. Stone J. L. Duvall J. A. Perederiy J. V. Bomar J. M. (2008). Linkage, association, and gene-expression analyses identify CNTNAP2 as an autism-susceptibility gene. *Am. J. Hum. Genet.* 82 150–159. 10.1016/j.ajhg.2007.09.005 18179893 PMC2253955

[B3] Albrecht U. Sutcliffe J. S. Cattanach B. M. Beechey C. V. Armstrong D. Eichele G. (1997). Imprinted expression of the murine Angelman syndrome gene, Ube3a, in hippocampal and Purkinje neurons. *Nat. Genet.* 17 75–78. 10.1038/ng0997-75 9288101

[B4] Amaral D. G. Schumann C. M. Nordahl C. W. (2008). Neuroanatomy of autism. *Trends Neurosci.* 31 137–145. 18258309 10.1016/j.tins.2007.12.005

[B5] Amegandjin C. A. Choudhury M. Jadhav V. Carrico J. N. Quintal A. Berryer M. (2021). Sensitive period for rescuing parvalbumin interneurons connectivity and social behavior deficits caused by TSC1 loss. *Nat. Commun.* 12:3653. 10.1038/s41467-021-23939-7 34135323 PMC8209106

[B6] Arking D. E. Cutler D. J. Brune C. W. Teslovich T. M. West K. Ikeda M. (2008). A common genetic variant in the neurexin superfamily member CNTNAP2 increases familial risk of autism. *Am. J. Hum. Genet.* 82 160–164. 10.1016/j.ajhg.2007.09.015 18179894 PMC2253968

[B7] Bahl S. Chiang C. Beauchamp R. L. Neale B. M. Daly M. J. Gusella J. F. (2013). Lack of association of rare functional variants in TSC1/TSC2 genes with autism spectrum disorder. *Mol. Autism* 4:5. 10.1186/2040-2392-4-5 23514105 PMC3610211

[B8] Bakkaloglu B. O’Roak B. J. Louvi A. Gupta A. R. Abelson J. F. Morgan T. M. (2008). Molecular cytogenetic analysis and resequencing of contactin associated protein-like 2 in autism spectrum disorders. *Am. J. Hum. Genet.* 82 165–173. 10.1016/j.ajhg.2007.09.017 18179895 PMC2253974

[B9] Bangash M. A. Park J. M. Melnikova T. Wang D. Jeon S. K. Lee D. (2011). Enhanced polyubiquitination of Shank3 and NMDA receptor in a mouse model of autism. *Cell* 145 758–772. 21565394 10.1016/j.cell.2011.03.052 PMC3110672

[B10] Baudouin S. J. Gaudias J. Gerharz S. Hatstatt L. Zhou K. Punnakkal P. (2012). Shared synaptic pathophysiology in syndromic and nonsyndromic rodent models of autism. *Science* 338 128–132. 10.1126/science.1224159 22983708

[B11] Bernier R. Golzio C. Xiong B. Stessman H. A. Coe B. P. Penn O. (2014). Disruptive CHD8 mutations define a subtype of autism early in development. *Cell* 158 263–276. 10.1016/j.cell.2014.06.017 24998929 PMC4136921

[B12] Bidinosti M. Botta P. Kruttner S. Proenca C. C. Stoehr N. Bernhard M. (2016). CLK2 inhibition ameliorates autistic features associated with SHANK3 deficiency. *Science* 351 1199–1203. 10.1126/science.aad5487 26847545

[B13] Bienvenu O. J. Needham D. M. Hopkins R. O. (2013). Diagnostic and statistical manual of mental disorders, Fifth Edition, and the impact of events scale-revised response. *Chest* 144 1974–1975. 10.1378/chest.13-1691 24297138

[B14] Bockers T. M. Segger-Junius M. Iglauer P. Bockmann J. Gundelfinger E. D. Kreutz M. R. (2004). Differential expression and dendritic transcript localization of Shank family members: identification of a dendritic targeting element in the 3’ untranslated region of Shank1 mRNA. *Mol. Cell. Neurosci.* 26 182–190. 10.1016/j.mcn.2004.01.009 15121189

[B15] Boeckers T. M. Kreutz M. R. Winter C. Zuschratter W. Smalla K. H. Sanmarti-Vila L. (1999). Proline-rich synapse-associated protein-1/cortactin binding protein 1 (ProSAP1/CortBP1) is a PDZ-domain protein highly enriched in the postsynaptic density. *J. Neurosci.* 19 6506–6518. 10414979 10.1523/JNEUROSCI.19-15-06506.1999 PMC6782800

[B16] Bonaglia M. C. Giorda R. Borgatti R. Felisari G. Gagliardi C. Selicorni A. (2001). Disruption of the ProSAP2 gene in a t(12;22)(q24.1;q13.3) is associated with the 22q13.3 deletion syndrome. *Am. J. Hum. Genet.* 69 261–268. 10.1086/321293 11431708 PMC1235301

[B17] Bourgeron T. (2015). From the genetic architecture to synaptic plasticity in autism spectrum disorder. *Nat. Rev. Neurosci.* 16 551–563. 10.1038/nrn3992 26289574

[B18] Canitano R. Palumbi R. (2021). Excitation/inhibition modulators in autism spectrum disorder: current clinical research. *Front. Neurosci.* 15:753274. 10.3389/fnins.2021.753274 34916897 PMC8669810

[B19] Cao W. Lin S. Xia Q. Q. Du Y. L. Yang Q. Zhang M. Y. (2018). Gamma oscillation dysfunction in mPFC leads to social deficits in neuroligin 3 R451C knockin mice. *Neuron* 97 1253.e7–1260.e7. 29503190 10.1016/j.neuron.2018.02.001

[B20] Cellot G. Cherubini E. (2014). GABAergic signaling as therapeutic target for autism spectrum disorders. *Front. Pediatr.* 2:70. 10.3389/fped.2014.00070 25072038 PMC4085902

[B21] Chahrour M. Jung S. Y. Shaw C. Zhou X. Wong S. T. Qin J. (2008). MeCP2, a key contributor to neurological disease, activates and represses transcription. *Science* 320 1224–1229. 10.1126/science.1153252 18511691 PMC2443785

[B22] Chang Q. Khare G. Dani V. Nelson S. Jaenisch R. (2006). The disease progression of Mecp2 mutant mice is affected by the level of BDNF expression. *Neuron* 49 341–348. 10.1016/j.neuron.2005.12.027 16446138

[B23] Chao H. T. Chen H. Samaco R. C. Xue M. Chahrour M. Yoo J. (2010). Dysfunction in GABA signalling mediates autism-like stereotypies and Rett syndrome phenotypes. *Nature* 468 263–269. 10.1038/nature09582 21068835 PMC3057962

[B24] Chen C. J. Sgritta M. Mays J. Zhou H. Lucero R. Park J. (2019). Therapeutic inhibition of mTORC2 rescues the behavioral and neurophysiological abnormalities associated with Pten-deficiency. *Nat. Med.* 25 1684–1690. 10.1038/s41591-019-0608-y 31636454 PMC7082835

[B25] Chen W. G. Chang Q. Lin Y. Meissner A. West A. E. Griffith E. C. (2003). Derepression of BDNF transcription involves calcium-dependent phosphorylation of MeCP2. *Science* 302 885–889. 10.1126/science.1086446 14593183

[B26] Chung C. Shin W. Kim E. (2021). Early and late corrections in mouse models of autism spectrum disorder. *Biol. Psychiatry* 91 934–944. 10.1016/j.biopsych.2021.07.021 34556257

[B27] Collins A. L. Levenson J. M. Vilaythong A. P. Richman R. Armstrong D. L. Noebels J. L. (2004). Mild overexpression of MeCP2 causes a progressive neurological disorder in mice. *Hum. Mol. Genet.* 13 2679–2689. 10.1093/hmg/ddh282 15351775

[B28] Cook E. H. Jr. Lindgren V. Leventhal B. L. Courchesne R. Lincoln A. Shulman C. (1997). Autism or atypical autism in maternally but not paternally derived proximal 15q duplication. *Am. J. Hum. Genet.* 60 928–934. 9106540 PMC1712464

[B29] Courchesne E. Mouton P. R. Calhoun M. E. Semendeferi K. Ahrens-Barbeau C. Hallet M. J. (2011). Neuron number and size in prefrontal cortex of children with autism. *J. Am. Med. Assoc.* 306 2001–2010. 10.1001/jama.2011.1638 22068992

[B30] Curran L. K. Newschaffer C. J. Lee L. C. Crawford S. O. Johnston M. V. Zimmerman A. W. (2007). Behaviors associated with fever in children with autism spectrum disorders. *Pediatrics* 120 e1386–e1392. 10.1542/peds.2007-0360 18055656

[B31] De Rubeis S. He X. Goldberg A. P. Poultney C. S. Samocha K. Cicek A. E. (2014). Synaptic, transcriptional and chromatin genes disrupted in autism. *Nature* 515 209–215. 10.1038/nature13772 25363760 PMC4402723

[B32] Durak O. Gao F. Kaeser-Woo Y. J. Rueda R. Martorell A. J. Nott A. (2016). Chd8 mediates cortical neurogenesis via transcriptional regulation of cell cycle and Wnt signaling. *Nat. Neurosci.* 19 1477–1488. 10.1038/nn.4400 27694995 PMC5386887

[B33] Durand C. M. Betancur C. Boeckers T. M. Bockmann J. Chaste P. Fauchereau F. (2007). Mutations in the gene encoding the synaptic scaffolding protein SHANK3 are associated with autism spectrum disorders. *Nat. Genet.* 39 25–27. 10.1038/ng1933 17173049 PMC2082049

[B34] Ehlers M. D. (2002). Molecular morphogens for dendritic spines. *Trends Neurosci.* 25 64–67. 10.1016/s0166-2236(02)02061-1 11814549

[B35] Ellenbroek B. Youn J. (2016). Rodent models in neuroscience research: is it a rat race? *Dis. Models Mech.* 9 1079–1087. 10.1242/dmm.026120 27736744 PMC5087838

[B36] Etherton M. R. Blaiss C. A. Powell C. M. Sudhof T. C. (2009). Mouse neurexin-1alpha deletion causes correlated electrophysiological and behavioral changes consistent with cognitive impairments. *Proc. Natl. Acad. Sci. U.S.A.* 106 17998–18003. 10.1073/pnas.0910297106 19822762 PMC2764944

[B37] Filice F. Vorckel K. J. Sungur A. O. Wohr M. Schwaller B. (2016). Reduction in parvalbumin expression not loss of the parvalbumin-expressing GABA interneuron subpopulation in genetic parvalbumin and shank mouse models of autism. *Mol. Brain* 9:10. 10.1186/s13041-016-0192-8 26819149 PMC4729132

[B38] Gauthier J. Champagne N. Lafreniere R. G. Xiong L. Spiegelman D. Brustein E. (2010). De novo mutations in the gene encoding the synaptic scaffolding protein SHANK3 in patients ascertained for schizophrenia. *Proc. Natl. Acad. Sci. U.S.A.* 107 7863–7868. 10.1073/pnas.0906232107 20385823 PMC2867875

[B39] Gogolla N. Takesian A. E. Feng G. Fagiolini M. Hensch T. K. (2014). Sensory integration in mouse insular cortex reflects GABA circuit maturation. *Neuron* 83 894–905. 10.1016/j.neuron.2014.06.033 25088363 PMC4177076

[B40] Gottfried C. Bambini-Junior V. Francis F. Riesgo R. Savino W. (2015). The impact of neuroimmune alterations in autism spectrum disorder. *Front. Psychiatry* 6:121. 10.3389/fpsyt.2015.00121 26441683 PMC4563148

[B41] Greer P. L. Hanayama R. Bloodgood B. L. Mardinly A. R. Lipton D. M. Flavell S. W. (2010). The Angelman Syndrome protein Ube3A regulates synapse development by ubiquitinating arc. *Cell* 140 704–716. 10.1016/j.cell.2010.01.026 20211139 PMC2843143

[B42] Guy J. Gan J. Selfridge J. Cobb S. Bird A. (2007). Reversal of neurological defects in a mouse model of Rett syndrome. *Science* 315 1143–1147. 10.1126/science.1138389 17289941 PMC7610836

[B43] He J. L. Oeltzschner G. Mikkelsen M. Deronda A. Harris A. D. Crocetti D. (2021). Region-specific elevations of glutamate + glutamine correlate with the sensory symptoms of autism spectrum disorders. *Transl. Psychiatry* 11:411. 10.1038/s41398-021-01525-1 34326312 PMC8322079

[B44] Heindel J. J. McAllister K. A. Worth L. Jr. Tyson F. L. (2006). Environmental epigenomics, imprinting and disease susceptibility. *Epigenetics* 1 1–6. 10.4161/epi.1.1.2642 17998808

[B45] Hering H. Sheng M. (2001). Dendritic spines: structure, dynamics and regulation. *Nat. Rev. Neurosci.* 2 880–888. 10.1038/35104061 11733795

[B46] Hogart A. Nagarajan R. P. Patzel K. A. Yasui D. H. Lasalle J. M. (2007). 15q11-13 GABAA receptor genes are normally biallelically expressed in brain yet are subject to epigenetic dysregulation in autism-spectrum disorders. *Hum. Mol. Genet.* 16 691–703. 10.1093/hmg/ddm014 17339270 PMC1934608

[B47] Hsiao E. Y. (2013). Immune dysregulation in autism spectrum disorder. *Int. Rev. Neurobiol.* 113 269–302. 24290389 10.1016/B978-0-12-418700-9.00009-5

[B48] Jamain S. Quach H. Betancur C. Rastam M. Colineaux C. Gillberg I. C. (2003). Mutations of the X-linked genes encoding neuroligins NLGN3 and NLGN4 are associated with autism. *Nat. Genet.* 34 27–29. 10.1038/ng1136 12669065 PMC1925054

[B49] Katayama Y. Nishiyama M. Shoji H. Ohkawa Y. Kawamura A. Sato T. (2016). CHD8 haploinsufficiency results in autistic-like phenotypes in mice. *Nature* 537 675–679. 10.1038/nature19357 27602517

[B50] Kemper T. L. Bauman M. (1998). Neuropathology of infantile autism. *J. Neuropathol. Exp. Neurol.* 57 645–652. 9690668 10.1097/00005072-199807000-00001

[B51] Kreienkamp H. J. Zitzer H. Gundelfinger E. D. Richter D. Bockers T. M. (2000). The calcium-independent receptor for alpha-latrotoxin from human and rodent brains interacts with members of the ProSAP/SSTRIP/Shank family of multidomain proteins. *J. Biol. Chem.* 275 32387–32390. 10.1074/jbc.C000490200 10964907

[B52] Krishnan V. Stoppel D. C. Nong Y. Johnson M. A. Nadler M. J. Ozkaynak E. (2017). Autism gene Ube3a and seizures impair sociability by repressing VTA Cbln1. *Nature* 543 507–512. 10.1038/nature21678 28297715 PMC5364052

[B53] Kwon C. H. Luikart B. W. Powell C. M. Zhou J. Matheny S. A. Zhang W. (2006). Pten regulates neuronal arborization and social interaction in mice. *Neuron* 50 377–388. 10.1016/j.neuron.2006.03.023 16675393 PMC3902853

[B54] Lewis M. H. Tanimura Y. Lee L. W. Bodfish J. W. (2007). Animal models of restricted repetitive behavior in autism. *Behav. Brain Res.* 176 66–74. 10.1016/j.bbr.2006.08.023 16997392 PMC3709864

[B55] Lim S. Sala C. Yoon J. Park S. Kuroda S. Sheng M. (2001). Sharpin, a novel postsynaptic density protein that directly interacts with the shank family of proteins. *Mol. Cell. Neurosci.* 17 385–397. 10.1006/mcne.2000.0940 11178875

[B56] Lu C. Chen Q. Zhou T. Bozic D. Fu Z. Pan J. Q. (2016). Micro-electrode array recordings reveal reductions in both excitation and inhibition in cultured cortical neuron networks lacking Shank3. *Mol. Psychiatry* 21 159–168. 10.1038/mp.2015.173 26598066

[B57] Luo J. Manning B. D. Cantley L. C. (2003). Targeting the PI3K-Akt pathway in human cancer: rationale and promise. *Cancer Cell* 4 257–262. 10.1016/s1535-6108(03)00248-4 14585353

[B58] Maenner M. J. Shaw K. A. Bakian A. V. Bilder D. A. Durkin M. S. Esler A. (2021). Prevalence and characteristics of autism spectrum disorder among children aged 8 years - autism and developmental disabilities monitoring network, 11 sites, United States, 2018. *MMWR Surveil. Summar.* 70 1–15. 10.15585/mmwr.ss7011a1 34855725 PMC8639024

[B59] Makedonski K. Abuhatzira L. Kaufman Y. Razin A. Shemer R. (2005). MeCP2 deficiency in Rett syndrome causes epigenetic aberrations at the PWS/AS imprinting center that affects UBE3A expression. *Hum. Mol. Genet.* 14 1049–1058. 10.1093/hmg/ddi097 15757975

[B60] Margolis S. S. Salogiannis J. Lipton D. M. Mandel-Brehm C. Wills Z. P. Mardinly A. R. (2010). EphB-mediated degradation of the RhoA GEF Ephexin5 relieves a developmental brake on excitatory synapse formation. *Cell* 143 442–455. 10.1016/j.cell.2010.09.038 21029865 PMC2967209

[B61] Martella G. Meringolo M. Trobiani L. De Jaco A. Pisani A. Bonsi P. (2018). The neurobiological bases of autism spectrum disorders: the R451C-neuroligin 3 mutation hampers the expression of long-term synaptic depression in the dorsal striatum. *Eur. J. Neurosci.* 47 701–708. 10.1111/ejn.13705 28921757

[B62] Martinowich K. Hattori D. Wu H. Fouse S. He F. Hu Y. (2003). DNA methylation-related chromatin remodeling in activity-dependent BDNF gene regulation. *Science* 302 890–893. 10.1126/science.1090842 14593184

[B63] Mei Y. Monteiro P. Zhou Y. Kim J. A. Gao X. Fu Z. (2016). Adult restoration of Shank3 expression rescues selective autistic-like phenotypes. *Nature* 530 481–484. 10.1038/nature16971 26886798 PMC4898763

[B64] Moretti P. Bouwknecht J. A. Teague R. Paylor R. Zoghbi H. Y. (2005). Abnormalities of social interactions and home-cage behavior in a mouse model of Rett syndrome. *Hum. Mol. Genet.* 14 205–220. 10.1093/hmg/ddi016 15548546

[B65] Moy S. S. Nadler J. J. Poe M. D. Nonneman R. J. Young N. B. Koller B. H. (2008). Development of a mouse test for repetitive, restricted behaviors: relevance to autism. *Behav. Brain Res.* 188 178–194. 10.1016/j.bbr.2007.10.029 18068825 PMC2349090

[B66] Naisbitt S. Kim E. Tu J. C. Xiao B. Sala C. Valtschanoff J. (1999). Shank, a novel family of postsynaptic density proteins that binds to the NMDA receptor/PSD-95/GKAP complex and cortactin. *Neuron* 23 569–582. 10.1016/s0896-6273(00)80809-0 10433268

[B67] Nan X. Campoy F. J. Bird A. (1997). MeCP2 is a transcriptional repressor with abundant binding sites in genomic chromatin. *Cell* 88 471–481. 10.1016/s0092-8674(00)81887-5 9038338

[B68] Nawaz Z. Lonard D. M. Smith C. L. Lev-Lehman E. Tsai S. Y. Tsai M. J. (1999). The Angelman syndrome-associated protein, E6-AP, is a coactivator for the nuclear hormone receptor superfamily. *Mol. Cell. Biol.* 19 1182–1189. 10.1128/MCB.19.2.1182 9891052 PMC116047

[B69] Numis A. L. Major P. Montenegro M. A. Muzykewicz D. A. Pulsifer M. B. Thiele E. A. (2011). Identification of risk factors for autism spectrum disorders in tuberous sclerosis complex. *Neurology* 76 981–987. 10.1212/WNL.0b013e3182104347 21403110 PMC3271577

[B70] Orefice L. L. Mosko J. R. Morency D. T. Wells M. F. Tasnim A. Mozeika S. M. (2019). Targeting peripheral somatosensory neurons to improve tactile-related phenotypes in ASD models. *Cell* 178 867.e24–886.e24. 10.1016/j.cell.2019.07.024 31398341 PMC6704376

[B71] O’Roak B. J. Vives L. Girirajan S. Karakoc E. Krumm N. Coe B. P. (2012). Sporadic autism exomes reveal a highly interconnected protein network of de novo mutations. *Nature* 485 246–U136. 10.1038/nature10989 22495309 PMC3350576

[B72] Peca J. Feliciano C. Ting J. T. Wang W. Wells M. F. Venkatraman T. N. (2011). Shank3 mutant mice display autistic-like behaviours and striatal dysfunction. *Nature* 472 437–442. 10.1038/nature09965 21423165 PMC3090611

[B73] Peixoto R. T. Wang W. Croney D. M. Kozorovitskiy Y. Sabatini B. L. (2016). Early hyperactivity and precocious maturation of corticostriatal circuits in Shank3B(-/-) mice. *Nat. Neurosci.* 19 716–724. 10.1038/nn.4260 26928064 PMC4846490

[B74] Penagarikano O. Abrahams B. S. Herman E. I. Winden K. D. Gdalyahu A. Dong H. (2011). Absence of CNTNAP2 leads to epilepsy, neuronal migration abnormalities, and core autism-related deficits. *Cell* 147 235–246. 10.1016/j.cell.2011.08.040 21962519 PMC3390029

[B75] Penagarikano O. Lazaro M. T. Lu X. H. Gordon A. Dong H. Lam H. A. (2015). Exogenous and evoked oxytocin restores social behavior in the Cntnap2 mouse model of autism. *Sci. Transl. Med.* 7:271ra8. 10.1126/scitranslmed.3010257 25609168 PMC4498455

[B76] Pensado-Lopez A. Veiga-Rua S. Carracedo A. Allegue C. Sanchez L. (2020). Experimental models to study autism spectrum disorders: hiPSCs, rodents and *Zebrafish*. *Genes* 11:1376. 10.3390/genes11111376 33233737 PMC7699923

[B77] Poliak S. Gollan L. Martinez R. Custer A. Einheber S. Salzer J. L. (1999). Caspr2, a new member of the neurexin superfamily, is localized at the juxtaparanodes of myelinated axons and associates with K+ channels. *Neuron* 24 1037–1047. 10.1016/s0896-6273(00)81049-1 10624965

[B78] Rademacher S. Eickholt B. J. (2019). PTEN in Autism and neurodevelopmental disorders. *Cold Spring Harb. Perspect. Med.* 9:a036780. 10.1101/cshperspect.a036780 31427284 PMC6824399

[B79] Ramsey M. M. Adams M. M. Ariwodola O. J. Sonntag W. E. Weiner J. L. (2005). Functional characterization of des-IGF-1 action at excitatory synapses in the CA1 region of rat hippocampus. *J. Neurophysiol.* 94 247–254. 10.1152/jn.00768.2004 15985695

[B80] Rapanelli M. Williams J. B. Ma K. Yang F. Zhong P. Patel R. (2022). Targeting histone demethylase LSD1 for treatment of deficits in autism mouse models. *Mol. Psychiatry* [Epub ahead of print]. 10.1038/s41380-022-01508-8 35296809 PMC9477974

[B81] Reed M. D. Yim Y. S. Wimmer R. D. Kim H. Ryu C. Welch G. M. (2020). IL-17a promotes sociability in mouse models of neurodevelopmental disorders. *Nature* 577 249–253. 10.1038/s41586-019-1843-6 31853066 PMC8112727

[B82] Reissner C. Klose M. Fairless R. Missler M. (2008). Mutational analysis of the neurexin/neuroligin complex reveals essential and regulatory components. *Proc. Natl. Acad. Sci. U.S.A.* 105 15124–15129. 10.1073/pnas.0801639105 18812509 PMC2551626

[B83] Ronan J. L. Wu W. Crabtree G. R. (2013). From neural development to cognition: unexpected roles for chromatin. *Nat. Rev. Genet.* 14 347–359. 10.1038/nrg3413 23568486 PMC4010428

[B84] Rotaru D. C. van Woerden G. M. Wallaard I. Elgersma Y. (2018). Adult Ube3a gene reinstatement restores the electrophysiological deficits of prefrontal cortex layer 5 neurons in a mouse model of angelman syndrome. *J. Neurosci.* 38 8011–8030. 10.1523/JNEUROSCI.0083-18.2018 30082419 PMC6596147

[B85] Rubenstein J. L. Merzenich M. M. (2003). Model of autism: increased ratio of excitation/inhibition in key neural systems. *Genes Brain Behav.* 2 255–267. 14606691 10.1034/j.1601-183x.2003.00037.x PMC6748642

[B86] Rylaarsdam L. Guemez-Gamboa A. (2019). Genetic causes and modifiers of autism spectrum disorder. *Front. Cell. Neurosci.* 13:385. 10.3389/fncel.2019.00385 31481879 PMC6710438

[B87] Sacai H. Sakoori K. Konno K. Nagahama K. Suzuki H. Watanabe T. (2020). Autism spectrum disorder-like behavior caused by reduced excitatory synaptic transmission in pyramidal neurons of mouse prefrontal cortex. *Nat. Commun.* 11:5140. 10.1038/s41467-020-18861-3 33046712 PMC7552417

[B88] Sala C. Piech V. Wilson N. R. Passafaro M. Liu G. Sheng M. (2001). Regulation of dendritic spine morphology and synaptic function by Shank and Homer. *Neuron* 31 115–130. 10.1016/s0896-6273(01)00339-7 11498055

[B89] Samaco R. C. Hogart A. LaSalle J. M. (2005). Epigenetic overlap in autism-spectrum neurodevelopmental disorders: MECP2 deficiency causes reduced expression of UBE3A and GABRB3. *Hum. Mol. Genet.* 14 483–492. 10.1093/hmg/ddi045 15615769 PMC1224722

[B90] Scattoni M. L. Crawley J. Ricceri L. (2009). Ultrasonic vocalizations: a tool for behavioural phenotyping of mouse models of neurodevelopmental disorders. *Neurosci. Biobehav. Rev.* 33 508–515. 10.1016/j.neubiorev.2008.08.003 18771687 PMC2688771

[B91] Scheffner M. Huibregtse J. M. Vierstra R. D. Howley P. M. (1993). The HPV-16 E6 and E6-AP complex functions as a ubiquitin-protein ligase in the ubiquitination of p53. *Cell* 75 495–505. 10.1016/0092-8674(93)90384-3 8221889

[B92] Shcheglovitov A. Shcheglovitova O. Yazawa M. Portmann T. Shu R. Sebastiano V. (2013). SHANK3 and IGF1 restore synaptic deficits in neurons from 22q13 deletion syndrome patients. *Nature* 503 267–271. 10.1038/nature12618 24132240 PMC5559273

[B93] Sheng M. Hoogenraad C. C. (2007). The postsynaptic architecture of excitatory synapses: a more quantitative view. *Annu. Rev. Biochem.* 76 823–847. 10.1146/annurev.biochem.76.060805.160029 17243894

[B94] Smith S. E. Zhou Y. D. Zhang G. Jin Z. Stoppel D. C. Anderson M. P. (2011). Increased gene dosage of Ube3a results in autism traits and decreased glutamate synaptic transmission in mice. *Sci. Transl. Med.* 3:103ra97. 10.1126/scitranslmed.3002627 21974935 PMC3356696

[B95] Smogavec M. Cleall A. Hoyer J. Lederer D. Nassogne M. C. Palmer E. E. (2016). Eight further individuals with intellectual disability and epilepsy carrying bi-allelic CNTNAP2 aberrations allow delineation of the mutational and phenotypic spectrum. *J. Med. Genet.* 53 820–827. 10.1136/jmedgenet-2016-103880 27439707

[B96] Sparks B. F. Friedman S. D. Shaw D. W. Aylward E. H. Echelard D. Artru A. A. (2002). Brain structural abnormalities in young children with autism spectrum disorder. *Neurology* 59 184–192. 10.1212/wnl.59.2.184 12136055

[B97] Sun J. Zhu G. Liu Y. Standley S. Ji A. Tunuguntla R. (2015). UBE3A regulates synaptic plasticity and learning and memory by controlling SK2 channel endocytosis. *Cell Rep.* 12 449–461. 10.1016/j.celrep.2015.06.023 26166566 PMC4520703

[B98] Swanberg S. E. Nagarajan R. P. Peddada S. Yasui D. H. LaSalle J. M. (2009). Reciprocal co-regulation of EGR2 and MECP2 is disrupted in Rett syndrome and autism. *Hum. Mol. Genet.* 18 525–534. 10.1093/hmg/ddn380 19000991 PMC2638799

[B99] Takeuchi K. Gertner M. J. Zhou J. Parada L. F. Bennett M. V. Zukin R. S. (2013). Dysregulation of synaptic plasticity precedes appearance of morphological defects in a Pten conditional knockout mouse model of autism. *Proc. Natl. Acad. Sci. U.S.A.* 110 4738–4743. 10.1073/pnas.1222803110 23487788 PMC3607034

[B100] Tanaka M. DeLorey T. M. Delgado-Escueta A. Olsen R. W. (2012). “GABRB3, epilepsy, and neurodevelopment,” in *Jasper’s Basic Mechanisms of the Epilepsies*, eds Noebels J. L. Avoli M. Rogawski M. A. Olsen R. W. Delgado-Escueta A. V. (Bethesda, MD: Oxford University Press). 39637091

[B101] Tate P. Skarnes W. Bird A. (1996). The methyl-CpG binding protein MeCP2 is essential for embryonic development in the mouse. *Nat. Genet.* 12 205–208. 10.1038/ng0296-205 8563762

[B102] Thompson B. A. Tremblay V. Lin G. Bochar D. A. (2008). CHD8 is an ATP-dependent chromatin remodeling factor that regulates beta-catenin target genes. *Mol. Cell. Biol.* 28 3894–3904. 10.1128/MCB.00322-08 18378692 PMC2423111

[B103] Tobaben S. Sudhof T. C. Stahl B. (2000). The G protein-coupled receptor CL1 interacts directly with proteins of the Shank family. *J. Biol. Chem.* 275 36204–36210. 10.1074/jbc.M006448200 10958799

[B104] Tropea D. Giacometti E. Wilson N. R. Beard C. McCurry C. Fu D. D. (2009). Partial reversal of Rett Syndrome-like symptoms in MeCP2 mutant mice. *Proc. Natl. Acad. Sci. U.S.A.* 106 2029–2034. 10.1073/pnas.0812394106 19208815 PMC2644158

[B105] Tsai P. T. Hull C. Chu Y. Greene-Colozzi E. Sadowski A. R. Leech J. M. (2012). Autistic-like behaviour and cerebellar dysfunction in Purkinje cell Tsc1 mutant mice. *Nature* 488 647–651. 10.1038/nature11310 22763451 PMC3615424

[B106] Tu J. C. Xiao B. Naisbitt S. Yuan J. P. Petralia R. S. Brakeman P. (1999). Coupling of mGluR/Homer and PSD-95 complexes by the Shank family of postsynaptic density proteins. *Neuron* 23 583–592. 10.1016/s0896-6273(00)80810-7 10433269

[B107] Uhlmann E. J. Apicelli A. J. Baldwin R. L. Burke S. P. Bajenaru M. L. Onda H. (2002). Heterozygosity for the tuberous sclerosis complex (TSC) gene products results in increased astrocyte numbers and decreased p27-Kip1 expression in TSC2+/- cells. *Oncogene* 21 4050–4059. 10.1038/sj.onc.1205435 12037687

[B108] van Slegtenhorst M. de Hoogt R. Hermans C. Nellist M. Janssen B. Verhoef S. (1997). Identification of the tuberous sclerosis gene TSC1 on chromosome 9q34. *Science* 277 805–808. 10.1126/science.277.5327.805 9242607

[B109] Verpelli C. Dvoretskova E. Vicidomini C. Rossi F. Chiappalone M. Schoen M. (2011). Importance of Shank3 protein in regulating metabotropic glutamate receptor 5 (mGluR5) expression and signaling at synapses. *J. Biol. Chem.* 286 34839–34850. 10.1074/jbc.M111.258384 21795692 PMC3186429

[B110] Voineagu I. Wang X. Johnston P. Lowe J. K. Tian Y. Horvath S. (2011). Transcriptomic analysis of autistic brain reveals convergent molecular pathology. *Nature* 474 380–384. 10.1038/nature10110 21614001 PMC3607626

[B111] Wang H. Chan S. A. Ogier M. Hellard D. Wang Q. Smith C. (2006). Dysregulation of brain-derived neurotrophic factor expression and neurosecretory function in Mecp2 null mice. *J. Neurosci.* 26 10911–10915. 10.1523/JNEUROSCI.1810-06.2006 17050729 PMC6674736

[B112] Wang X. Bey A. L. Katz B. M. Badea A. Kim N. David L. K. (2016). Altered mGluR5-Homer scaffolds and corticostriatal connectivity in a Shank3 complete knockout model of autism. *Nat. Commun.* 7:11459. 10.1038/ncomms11459 27161151 PMC4866051

[B113] Wang X. McCoy P. A. Rodriguiz R. M. Pan Y. Je H. S. Roberts A. C. (2011). Synaptic dysfunction and abnormal behaviors in mice lacking major isoforms of Shank3. *Hum. Mol. Genet.* 20 3093–3108. 10.1093/hmg/ddr212 21558424 PMC3131048

[B114] Wohr M. Scattoni M. L. (2013). Behavioural methods used in rodent models of autism spectrum disorders: current standards and new developments. *Behav. Brain Res.* 251 5–17. 10.1016/j.bbr.2013.05.047 23769995

[B115] Yang M. Silverman J. L. Crawley J. N. (2011). Automated three-chambered social approach task for mice. *Curr. Protoc. Neurosci.* 56 8.26.1–8.26.16. 10.1002/0471142301.ns0826s56 21732314 PMC4904775

[B116] Yao I. Hata Y. Hirao K. Deguchi M. Ide N. Takeuchi M. (1999). Synamon, a novel neuronal protein interacting with synapse-associated protein 90/postsynaptic density-95-associated protein. *J. Biol. Chem.* 274 27463–27466. 10.1074/jbc.274.39.27463 10488079

[B117] Yi F. Danko T. Botelho S. C. Patzke C. Pak C. Wernig M. (2016). Autism-associated SHANK3 haploinsufficiency causes Ih channelopathy in human neurons. *Science* 352:aaf2669. 10.1126/science.aaf2669 26966193 PMC4901875

[B118] Yoo H. (2015). Genetics of autism spectrum disorder: current status and possible clinical applications. *Exp. Neurobiol.* 24 257–272. 10.5607/en.2015.24.4.257 26713075 PMC4688327

[B119] Zaccaria K. J. Lagace D. C. Eisch A. J. McCasland J. S. (2010). Resistance to change and vulnerability to stress: autistic-like features of GAP43-deficient mice. *Genes Brain Behav.* 9 985–996. 10.1111/j.1601-183X.2010.00638.x 20707874 PMC2975747

[B120] Zappella M. Meloni I. Longo I. Hayek G. Renieri A. (2001). Preserved speech variants of the Rett syndrome: molecular and clinical analysis. *Am. J. Med. Genet.* 104 14–22. 10.1002/ajmg.10005 11746022

[B121] Zeng L. H. Xu L. Gutmann D. H. Wong M. (2008). Rapamycin prevents epilepsy in a mouse model of tuberous sclerosis complex. *Ann. Neurol.* 63 444–453. 10.1002/ana.21331 18389497 PMC3937593

[B122] Zheng W. H. Quirion R. (2004). Comparative signaling pathways of insulin-like growth factor-1 and brain-derived neurotrophic factor in hippocampal neurons and the role of the PI3 kinase pathway in cell survival. *J. Neurochem.* 89 844–852. 10.1111/j.1471-4159.2004.02350.x 15140184

[B123] Zhou J. Blundell J. Ogawa S. Kwon C. H. Zhang W. Sinton C. (2009). Pharmacological inhibition of mTORC1 suppresses anatomical, cellular, and behavioral abnormalities in neural-specific Pten knock-out mice. *J. Neurosci.* 29 1773–1783. 10.1523/JNEUROSCI.5685-08.2009 19211884 PMC3904448

[B124] Zhou J. Parada L. F. (2009). A motor driving PTEN. *Nat. Cell Biol.* 11 1177–1179. 10.1038/ncb1009-1177 19794503

[B125] Zitzer H. Honck H. H. Bachner D. Richter D. Kreienkamp H. J. (1999a). Somatostatin receptor interacting protein defines a novel family of multidomain proteins present in human and rodent brain. *J. Biol. Chem.* 274 32997–33001. 10.1074/jbc.274.46.32997 10551867

[B126] Zitzer H. Richter D. Kreienkamp H. J. (1999b). Agonist-dependent interaction of the rat somatostatin receptor subtype 2 with cortactin-binding protein 1. *J. Biol. Chem.* 274 18153–18156. 10.1074/jbc.274.26.18153 10373412

